# Influence of admission categories, programme, race and residence status on MBBCh and BHSc student performance: Academic performance of 2021 students in physiology module

**DOI:** 10.4102/hsag.v30i0.2933

**Published:** 2025-11-24

**Authors:** Sfiso E. Mabizela, Eliton Chivandi

**Affiliations:** 1 Centre for Health Science Education, Faculty of Health Sciences, University of the Witwatersrand, Johannesburg, South Africa; 2 School of Physiology, Faculty of Health Sciences, University of the Witwatersrand, Johannesburg, South Africa

**Keywords:** academic performance, admission categories, health education, health sciences, physiology

## Abstract

**Background:**

The Medical Physiology and Biochemistry (PHSL 2004A) course is a compulsory 2nd-year requirement for the Bachelor of Medicine and Bachelor of Surgery (MBBCH) and Bachelor of Health Sciences (BHSc) programmes. A significant decline in pass rates was observed among the 2020 cohort assessed in 2021.

**Aim:**

This study aimed to investigate the influence of programme affiliation, residence status (on-campus or off-campus), admission category and race on PHSL 2004A pass rates in 2021.

**Setting:**

The study was conducted at the University of the Witwatersrand, focusing on Faculty of Health Sciences students.

**Methods:**

A quantitative approach was adopted, analysing data from 380 students (218 MBBCh; 162 BHSc). Independent samples *t*-tests, one-way between-groups ANOVA and chi-square tests were employed for analysis.

**Results:**

MBBCh students achieved higher average scores (*M* = 60.76) compared to BHSc students (*M* = 47.98), demonstrating better overall performance in the course. Students living off-campus showed slightly higher pass rates (72%) than those residing on-campus (65%), although this difference was statistically insignificant. Admission category played a significant role in performance, with students admitted under the Top 40 category achieving higher scores than those admitted under the top rural and top BC categories.

**Conclusion:**

Programme affiliation and admission category significantly influenced PHSL 2004A performance, whereas residential status did not exhibit a notable effect.

**Contribution:**

This study highlights key predictors of academic performance in PHSL 2004A, offering insights that can inform targeted interventions to improve pass rates and support underperforming student groups.

## Introduction

The global emergence of the coronavirus disease 2019 (COVID-19) pandemic has precipitated profound transformations within the landscape of higher education on a worldwide scale. In response to the exigencies posed by the pandemic, educational institutions have been compelled to expeditiously reconfigure their operational frameworks, pedagogical methodologies and evaluative paradigms. Consequently, academic establishments, including the Faculty of Health Sciences at the University of the Witwatersrand, have expeditiously embraced the integration of online instructional modalities and the augmentation of digital infrastructural capacities to sustain educational continuity amid the prevailing challenges.

This study was conducted at the University of the Witwatersrand after it was noted that the academic performance of students enrolled in the 2nd-year course of Physiology and Medical Biochemistry (PHSL 2004A) at Wits University School of Physiology exhibited a decrease in performance in 2021. The decrease was noted among students enrolled in medical (MBBCh) and Bachelor of Health Science (BHSc) degree programmes offered by the Faculty of Health Sciences. In previous years, the pass rates for the MBBCh programme ranged from 82% to 98%, and for the BHSc programme, they ranged from 65% to 95%. In 2021, the pass rates were 77% for the MBBCh programme and 53% for the BHSc programme. In view of the drop in student performance in the PHSL 2004A course in the 2020 cohort, we analysed the decline in student performance by examining data based on admission categories, programme, race and residence status while acknowledging the influence of asynchronous teaching methods implemented in response to COVID-19 restrictions and the possible contribution of some factors that are known to influence students’ performance and progression in tertiary education.

Prospective students applying for the above programmes must take the National Benchmark Test (NBT), which accounts for 50% of the composite index used to determine eligibility. The remaining 50% is obtained from the combined results achieved in specific subjects of the National Senior Certificate, which is required for admission into the programme. Afterward, the composite index is used to organise applicants into four unique categories. The first category allocates 40% of available positions for the top-performing students, while the secondary category reserves 60% of allocations for high-performing candidates from rural areas, quintiles 1–2 and black or coloured students.

The National Benchmark Test 2020 report highlighted the need for the higher education sector to offer substantial support in quantitative literacy (QL) and mathematics (MAT), as a large majority of potential students achieved scores within the Basic and Intermediate benchmark ranges (CETAP 2020). This cohort was admitted during a time when the Faculty of Health Sciences used an admission policy that included the requirement for students to write NBT in addition to the National Senior Certificate (Merwe et al. 2015). The core domains assessed in the NBT for selection in health sciences programmes are Mathematics, Academic and Quantitative Literacy (Mabizela & George 2020). The critical subjects compulsory in the NSC for admission are Mathematics, English, Physical Sciences or Life Sciences.

### Literature review

Academic performance is vaguely defined as the success of a person in formal education (Banai & Perin 2016), often assessed by course performance (Conard 2006), 1st-year examination result (Busato et al. 2000), final grade point average (Goff & Ackerman 1992) or standardised test scores (Ii et al. 2021). Academic performance at the tertiary level has a strong bearing on one’s quality of life and mental, physical and socio-economic well-being (Hagerty et al. 2001). It affects the job application process and provides valuable information to prospective employers (Plant et al. 2005). Mostly, the onus of the students’ performance rests significantly on them (García & Elba 2021). However, the institution’s success is also partially rated by the academic performance of its students (Farooq et al. 2011; Pietrucha 2018). Thus, the students are the most valuable assets to the university (Farooq et al. 2011); hence, tertiary institutions put in place measures to enrol the best of students and mentor them appropriately to ensure sustained attainment of success.

Allocating 1st-year places to students in the university is always an issue for academic institutions (Cyrenne & Chan 2012). The decision to admit students into a particular course is based on several factors (Vista & Alkhadim 2022). Attaining a high grade in high school is a key determinant for gaining admission into high-demand courses (Vista & Alkhadim 2022), and MBBCh and BHSc courses are no exception. High school grades tend to correlate positively with academic success at the undergraduate level (Tumen, Shulruf & Hattie 2008); thus, it is the most common selective criterion used at entry points (Pitman 2016). Nevertheless, for these highly competitive courses, the utilisation of this criterion results in the over-representation of individuals from socio-economically privileged backgrounds who have attended selective schools (Tumen et al. 2008). For example, studies show that students from such affluent schools have the advantage of obtaining high scores in high school because of access to study material, requisite equipment and teachers with high educational backgrounds (McManus et al. 2013; Mwandigha et al. 2018). However, it has been shown that students from high-performing high schools tend to receive poorer grades at the tertiary level after controlling for high school grades (Mwandigha et al. 2018). Hence, to include students with intellectual ability and/or from less privileged backgrounds, many universities conduct standardised tests to normalise high school grades from different schools (Ii et al. 2021). The use of standardised tests has predictive validity for success in undergraduate programmes. Key determinants of students’ academic performance are not limited to high school grades and high scores on the standardised tests. Once enrolled, other factors also come into play in the students’ academic performance.

In 1953, the apartheid government introduced the *Bantu Education Act (Act No. 47 of 1953)* to entrench and deliver on the promise of separateness among races in South Africa (Anderson 2020; Louw et al. 2021; Low 1958). The Act included the creation of a Black Education Department which was responsible for implementing a curriculum aimed at equipping black individuals with basic skills to work for white people (Louw et al. 2021). The schools administered under the *Bantu Education Act* and inspected by the Union Department of Native Affairs were established in disadvantaged areas and offered an inferior education, a situation that persists to this day (Anderson 2020; Low 1958). The Bantu Education system has significantly restricted the ability of black, Indian and coloured individuals to attend historically white universities. The situation was further exacerbated by the *University Education Act of 1959*, which made it extremely challenging for certain racial groups to gain entry into historically white universities without obtaining high-level ministerial permission (Louw et al. 2021).

The democratic South African government introduced the school quintile system, implemented as a means to reverse the lasting legacy of the *Bantu Education Act*, including major curriculum revisions (Graven 2014). The quintile system categorises schools into five quintiles based on the socio-economic status of their surrounding communities, with Quintile 1 representing the most economically disadvantaged and Quintile 5 the most advantaged (Ogbonnaya & Awuah 2019). While ostensibly designed to address inequality by directing more resources to schools serving impoverished areas, this system has instead entrenched inequalities and marginalised students from lower quintile schools. Schools situated in wealthier areas (Quintiles 4 and 5) typically enjoy better infrastructure, resources and teaching quality, while those in lower quintiles struggle with inadequate facilities, insufficient staffing and limited access to educational materials and technologies (Ogbonnaya & Awuah 2019). Consequently, students attending lower quintile schools are disproportionately disadvantaged in terms of educational opportunities, resources and outcomes. Moreover, the quintile system has inadvertently created a two-tiered education system, with students from affluent backgrounds accessing higher-quality education and opportunities in Quintile 5 schools, while those from disadvantaged backgrounds face systemic barriers to academic achievement and socio-economic mobility in lower school quintiles (Walton, Bowman & Osman 2016). Thus, while the intention behind the quintile system may have been to address educational disparities, its implementation has ultimately reinforced inequalities between the rich and the poor, further widening the gap and hindering equitable access to quality education for all South African students (Ogbonnaya & Awuah 2019; Van Dyk & White 2019). Besides the already existing achievement gap between black and white students (Carnoy & García 2017), white–black teacher to student mismatch also affects academic performance (Egalite, Kisida & Winters 2015).

Although inconclusive, the accommodation status of students contributes substantially to academic performance, with off-campus accommodation showing an advantageous effect on academic performance (Mbandlwa 2022). This challenges the rationale of providing on-campus accommodation by universities to facilitate easy access to study amenities.

### Aims

The overall aim of this study was to understand the factors influencing academic performance and progression outcomes among MBBCh and BHSc students enrolled in the PHSL2004A course, with a particular focus on demographic variables, living arrangements, race and admission categories under the environment of COVID-19-induced asynchronous learning environment.

## Research methods and design

This study employed a quantitative approach, incorporating both parametric and non-parametric statistical analyses to explore factors influencing academic performance in the PHSL2004A course.

### Setting

The study was conducted at the University of the Witwatersrand in the Faculty of Health Sciences. The PHSL2004A course is a compulsory 2nd-year module for students enrolled in the Bachelor of Medicine and Bachelor of Surgery (MBBCh) and Bachelor of Health Sciences (BHSc) programmes.

### Study population and sampling

The study population consisted of 2nd-year MBBCh and BHSc students registered in the 2021 academic year. A total sample of 380 students (218 MBBCh and 162 BHSc) was analysed. The sample was derived from datasets provided by the School of Physiology administrator and the Business Intelligence Service. Four students were excluded from the analysis because of violations of the normality assumption, resulting in a final sample of 376 students.

### Data collection

Data were collected from two sources as mentioned above. To break down the data requested and received from the sources: the School of Physiology administrator provided data on student numbers, race, programme enrolment and end-of-year results for PHSL2004A. The Business Intelligence Service provided additional datasets on admission data, place of origin, school quintiles, living arrangements, race and progression outcomes. The datasets were matched using VLOOKUP, and all identifiable student data were removed before analysis to ensure anonymity.

### Data analysis

Data analysis was conducted using Statistical Package for Social Sciences (SPSS) (version 2023). The independent samples *t*-test was used to compare the mean scores between MBBCh and BHSc students to identify significant differences in performance (Allen, Bennett & Heritage 2014). The chi-square test assessed potential associations between academic outcomes and residential status or racial background, with progression outcomes coded as binary variables: pass ≥ 50%; fail ≤ 49% (Allen et al. 2014).

A one-way-between-groups ANOVA was conducted to compare mean scores across admission categories within and between the two programmes (Allen et al. 2014). Post-hoc analyses employed the Gabriel procedure to account for unequal group sizes (Allen et al. 2014). Assumptions for all statistical tests, including scale type, independence, normality and homogeneity of variance, were verified prior to analysis (Allen et al. 2014).

### Ethical considerations

This study received ethical clearance from the Human Research Ethics Committee (Medical) of the University of the Witwatersrand (approval number: M220561). The research utilised de-identified secondary data of students, ensuring no direct involvement of human participants. Informed consent was waived by the Human Research Ethics Committee because of the use of de-identified data, which safeguarded participant confidentiality and privacy. No identifying information was accessible or included in the analysis, and all measures adhered to the committee’s standards for maintaining data confidentiality. The study did not involve animal research, and the use of de-identified secondary data qualified it as non-human subjects’ research.

## Results

### Sample demographics

The study sample demographic characteristics are shown in Table 1 and represent all students prior to the removal of four students. Admission categories Top 40 and Top B and C constituted 84% of the sample. In terms of race and residence status, both black students and in-university residence students each contributed 51% of the sample. The MBBCh programme accounted for 57% of the sample population.

**TABLE 1:** Admission category, race, residence status and programme of the study sample (*N* = 380).

Parameter	Variable	*n*	Percentage
Admission category	Top 40	173	46
	Top Rural	48	13
	Top Quintile 1 and 2	16	4
	Top Black and Coloured students	143	38
Race	Black students	192	51
	White students	82	22
	Coloured students	23	6
	Indian students	83	22
Residence status	On campus	195	51
	Off campus	185	49
Programme	MBBCh	218	57
	BHSc	162	43
	Total	380	100

MBBCh, Bachelor of Medicine and Bachelor of Surgery; BHSc, Bachelor of Health Sciences.

### Independent sample *t*-test results, *N* = 376

The results of an independent sample *t*-test comparing MBBCh (*n* = 216) and BHSc (*n* = 160) students’ performance in the PHSL2004A were statistically significant, *p* ≤ 0.001. The MBBCh students (*M* = 60.76, standard deviation [s.d.] = 17.21) had a higher mean score of 12.70, 95% confidence interval (CI) [9.404, 15.990] than BHSc (*M* = 47.98, s.d. = 14.34), 95% CI [9.490, 15.902], *t* (374) = 7.58, *p* ≤ 0.001, two tailed, *d* = 0.79.

### Chi-square results, *N* = 376

**FIGURE 1:**
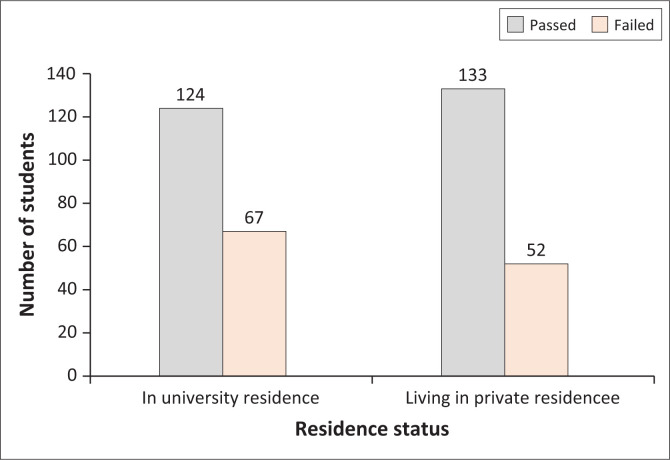
Students’ living arrangements by progression outcomes.

A chi-square test to analyse the association between students’ living arrangements and their progression outcome in the PHSL2004A was not a statistically significant association (χ^2^ = 2.111, degrees of freedom [*df*] = 1, *N* = 376, *p* < 0.146). Out of the 185 students residing in private residential areas, 28% (*n* = 52) encountered failure, while the remaining 72% (*n* = 133) achieved a passing outcome. On the other hand, out of the students residing on campus, 35% (*n* = 67) experienced failure, while 65% (*n* = 124) successfully passed the course. Upon further analysis of students’ race based on their residential arrangement, two main groups were identified among those living on campus. Black students accounted for 40.4% (*n* = 152) of students living on campus compared to white students, 5.9% (*n* = 22). On the other hand, the largest groups of students living in private residential areas were Indian students, comprising 18.6% (*n* = 70), and white students, comprising 16.0% (*n* = 60). Although the overall relationship between living arrangements and progression outcome may not have reached statistical significance, these findings do indicate possible patterns among certain racial groups in terms of their housing choices (see Figure 1 demonstrating progression patterns by students’ living arrangements).

**FIGURE 2:**
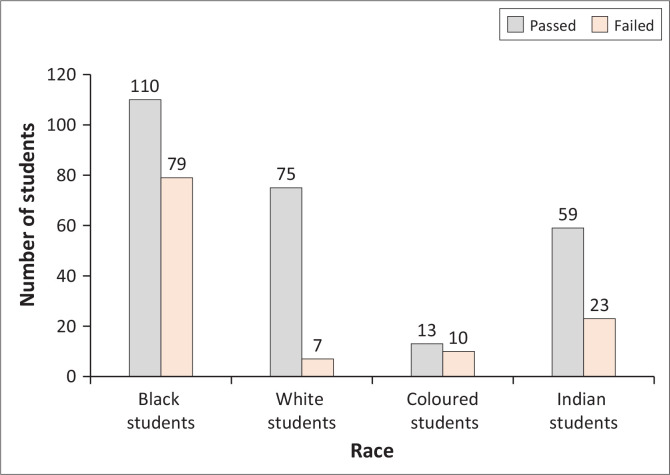
Race by students’ progression outcomes.

The chi-square test conducted to analyse the relationship between race description and progression outcomes yielded statistically significant results (χ^2^ = 31.229, *df* = 1, *N* = 376, *p* < 0.001). Upon examining the crosstabulation, it becomes evident that the distribution of progression outcomes differs among various racial groups. Out of the black students, 58.2% passed, while 41.8% were unsuccessful. In contrast, the passing rate for white students was significantly higher at 91.5%, while only a small proportion of 8.5% failed. Among coloured students, 56.5% passed, while 43.5% failed. Out of the Indian students, 72.0% passed, while 28.0% failed. In summary, the chi-square test demonstrates a significant association between race and progression outcome. However, it is important to emphasise that the chi-square test only reveals an association and does not establish causation (see Figure 2 illustrating progression patterns by race).

### ANOVA results for both MBBCh and BHSc, *N* = 376

**FIGURE 3:**
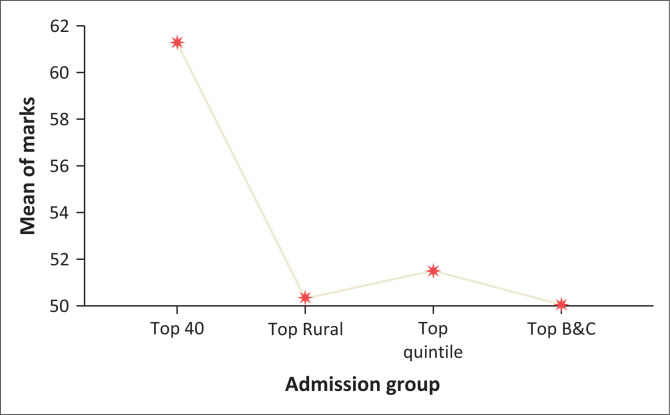
MBBCh and BHSc mean scores in the PHSL2004A.

The ANOVA results were statistically significant, suggesting that admission categories influenced both MBBCh and BHSc students’ academic performance in the PHSL2004A, *F* (3, 372) = 14.212, *p* = 0.001, Ƞ^2^ = 10. Post-hoc analysis using Gabriel’s procedure (α = 0.05) showed that students admitted to the top 40 categories (*M* = 61.27, s.d. = 17.01) had a higher mean score than top rural students (*M* = 50.30, s.d. = 16.20) with a mean difference of 10.96, which was statistically significant, *p* = 0.001. A statistically significant difference between the top 40 and top BC (*M* = 50.04, s.d. = 15.48) with a mean difference of 11.23, *p* = 0.001, was observed. No significant differences were observed between students admitted to the top 40 and quintiles 1–3 and the top rural and BC categories. Figure 3 shows the mean scores of MBBCh and BHSc by admission categories.

### BHSc, *N* = 160

**FIGURE 4:**
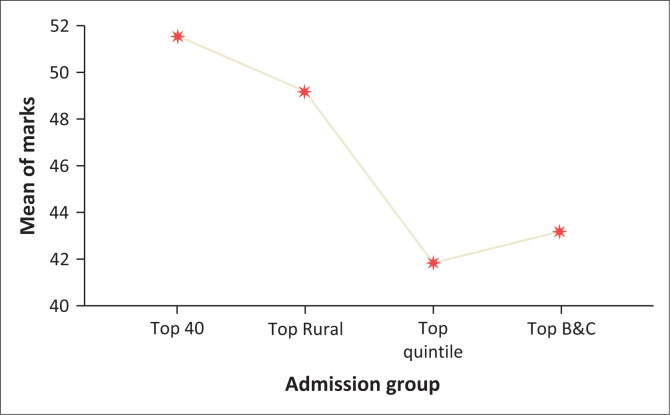
BHSc students’ mean scores in the PHSL2004A.

The ANOVA results were statistically significant, suggesting that admission categories explained BHSc students’ academic performance in the PHSL2004A, *F* (3, 156) = 4.671, *p* = 0.004, Ƞ^2^ = 08. The post-hoc analysis indicated that students in the top 40 (*M* = 51.56, s.d. = 14.66) performed better than students in the top BC category (*M* = 47.98, s.d. = 14.34) with a mean difference of 8.391, *p* = 0.002. No statistically significant differences were observed among other groups (see Figure 4 illustrating BHSc students’ mean scores by admission categories).

### MBBCh, *N* = 216

**FIGURE 5:**
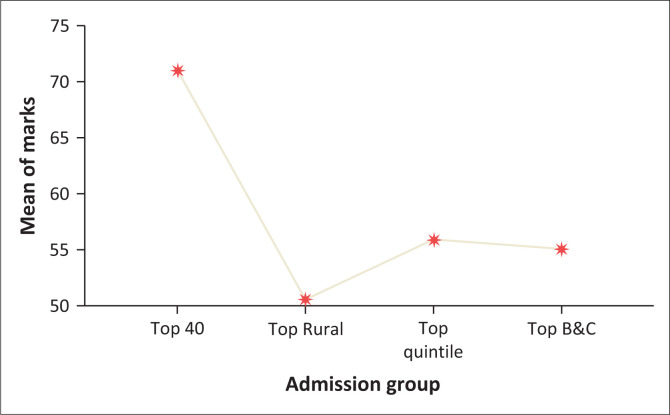
MBBCh students’ mean score in the PHSL2004A.

The ANOVA results were statistically significant, suggesting that admission categories explained MBBCh students’ academic performance in the PHSL2004A, *F* (3, 212) = 23.183, *p* = 0.001, Ƞ^2^ = 24. The post-hoc analysis revealed that students in the top 40 (*M* = 70.98, s.d. = 13.27) performed better than students in the top rural category (*M* = 50.57, s.d. = 16.46) with a mean difference of 20.40, *p* = 0.001. The students in the top 40 categories performed better than the top quintile students (*M* = 55.91, s.d. = 19.24), with a mean difference of 15.06, *p* = 0.004. Similar results were observed between the top 40 and top BC (*M* = 55.06, s.d. = 15.53), with a mean difference of 15.91, *p* = 0.001. No significant differences were observed when top rural, quintile and BC were compared with each other (see Figure 5 showing MBBCh students’ mean scores in PHSL2004A).

## Discussion

The study aimed to investigate various factors influencing academic performance and progression outcomes among medical (MBBCh) and health science (BHSc) students in the context of a specific physiology course (PHSL2004A). Through a comprehensive analysis encompassing independent sample *t*-tests, chi-square tests and ANOVA, the study explored the impact of demographic variables such as living arrangements, race and admission categories on students’ academic achievements and progression outcomes. The results of these analyses provided valuable insights into the complex interplay between individual characteristics and educational environments, shedding light on potential disparities and areas for targeted interventions within medical and health science education. In this discussion, we delve into the implications of these findings, addressing the significance of each factor in shaping student outcomes and proposing avenues for future research and educational practices aimed at enhancing academic success and equity within the medical and health science disciplines.

We observed that MBBCh programme students performed better with higher mean scores compared to their BHSc counterparts. The chi-square test of association revealed no statistically significant difference in the academic performance of PHSL 2004 students in 2021 based on their accommodation type, whether on-campus residences or off-campus private accommodations. However, existing research suggests that students residing in private accommodations often achieve better academic outcomes compared to those living in university residences, highlighting ongoing debates on the influence of accommodation on academic performance (Gichere, Adem & Adenya 2019; Mabizela & Bruce 2022; Mbandlwa 2022).

Meaningful academic performance has been shown to be dependent on a good housing environment that offers comfort, convenience, satisfaction and overall life fulfilment (Ghani & Suleiman 2016). A non-conducive student accommodation environment has been shown to increase the risk of developing health problems, stress and depression that negatively impact academic performance (Ghani & Suleiman 2016). Hence, the provision of an on-campus student accommodation that positively contributes to the enhancement of the overall curriculum environment is one of the priority developmental areas in higher education institutions (Adesoji & Jiboye 2010). The lack of association in the performance of students in the PHSL 2004 course suggests that both off-campus and on-campus student accommodation offered the same housing environment and perhaps amenities such that there was neither a disadvantage nor an advantage for students to be in either of the types of accommodation. However, although we did not find a statistically significant association in the performance of the off-campus and on-campus students in the 2021 PHSL 2004A course, 72% of the off-campus students compared to 65% of on-campus students passed the course. The higher percentage pass rate by off-campus accommodated students, although not statistically significant, corroborates the assertion by Mbandlwa (2022) that off-campus accommodation provides higher means in academic performance. When just considering the nominal difference in percentage of pass rate, our findings suggest that although on-campus accommodation provides for proximity to educational amenities, this may not necessarily advantage on-campus accommodated students, which makes one question the rationale of investing in on-campus accommodation, particularly with current advances in access to information, even via smartphones. However, a thorough evaluation of the socio-economic and support status that surround the off-campus accommodated students needs to be dissected before drawing definitive conclusions, as these may be drivers of better performance, especially in historically advantaged sections of society.

Findings from the current study show that 91% of white students passed the PHSL 2004A course in 2021 compared to 61% for the combined black, coloured and Indian students (Figure 1). Despite advancements in school transformation and increased access to education for historically disadvantaged communities, the lingering effects of apartheid, particularly the enduring impact of the discriminatory Bantu Education policies, persist, underscored by the deliberate dismantling of educational infrastructure that once served as the foundation for the political elite (Wilson 2011). In these communities, racial and ethnic inequality remains as one of the most significant social issues. The difference in pass per cent by race does not reflect any difference in intellectual capacity between the races but is a reflection of the effect of differences between ‘starting out’ from an ‘advantaged and privileged’ position compared to from a ‘disadvantaged and unprivileged’ platform. While we unreservedly subscribe to and agree with the fact that intellectual capacity is similar across human societies, we are also alive to and cognisant of the existence of an achievement gap between black and white students (Carnoy & García 2017); that, in our considered view, gives historically advantaged students a privileged foundation in academics, which is not a consequence of superior intellectual capacity but one of better access to resources. Further to the effect of resource-induced achievement gap between white people and black people, white–black teacher to student mismatch has also been observed to affect academic performance (Egalite et al. 2015).

In their study on determinants of academic performance in medical students, Ekwochi et al. (2019) report that mode of admission into medical school had the strongest correlation with good academic performance, making it a key predictor of good academic performance. Our study findings show that admission category affected both BHSc and MBBCh students’ performance in the PHSL 2004A course in 2021. Overall, students admitted in the Top 40 category had higher mean scores compared to their counterparts admitted in the Top rural category. On a programme basis, BHSc students admitted in the Top 40 category performed better than their counterparts admitted in the Top BC category, and MBBCh students admitted in the Top 40 category performed better compared to their counterparts admitted in theTop rural and Top BC categories, but there was no difference in performance for students admitted in the Top rural, quintile and Top BC categories. Our results are in tandem with the observation by Ekwochi et al. (2019). Admission category-dependent differences in student performance require medical schools to craft and implement special support programmes that target assisting students whose admission categories are and or have been associated with lower academic performance. While the aim of enrolling students on the basis of different admission categories could be to spread, open and improve access to medical science education to broader society, medical faculties must invest in targeted support schemes in order to increase and improve academic performance, especially of students from historically disadvantaged communities whose admission categories may be associated with lower academic performance.

The decreased pass rates (performance) in the PHSL 2004A course in 2021 by BHSc and MBBCh programme students occurred in a cohort that enrolled for 1st-year university education in 2020 during the global COVID-19 pandemic. During this period, governments imposed lockdowns in order to minimise human contact and contain the spread of the novel coronavirus, the cause of COVID-19 (Bonaccorsi et al. 2020). During the COVID-19 pandemic years 2020 to 2022, Wits University, like other higher education institutions, migrated from the traditional didactic face-to-face content delivery in a physical class to using the online platform to facilitate learning. This online mode of delivery tended to be more of an asynchronous online learning format where students listened to voice-over PowerPoint presentations and or viewed instructional material largely at the time of their choice. There was very little, if any, online synchronous engagement, which would have allowed students to participate in live online synchronous learning and/or engagement sessions. The heavy dependency on asynchronous online learning deprived students in the two programmes of both in-person and/or live online synchronous engagement. Previous research has shown that outcomes of online learning are largely mixed. In a study, Hurlbut (2018) reported that students who learnt in physical class settings performed better when compared to counterparts exposed to online content delivery. Furthermore, Sintema (2020) reported that students’ academic performance was significantly impacted by their presence and or absence from physical classes. Physical classes allow for in-person individual activities that are crucial to the students’ comprehension of the subject matter (Şen 2013) and that translate to better performance on assessment.

The pass rates of MBBCh and BHSc students in the PHSL 2004A course during the period 2013 to 2020 ranged from 82% to 98% and 65% to 95%, respectively. In 2021, the pass rates dropped to 77% and 53% for the MBBCh and BHSc programmes, respectively. This drop in student performance occurred in a period when knowledge construction by students was mediated largely through an online asynchronous mode. Our findings seem to confirm the observations and assertions by Hurlbut (2018) and Sintema (2020), who argued that students exposed to physical classes tend to perform better than those exposed to online learning. The authors are of the view that virtual online asynchronous content delivery may have deprived students of opportunities for in-person individual activities, which have been demonstrated to buttress students’ comprehension of subject matter content offered by and in physical classes (Şen 2013). Our findings suggest that the 2020 cohort of students experienced a significant loss in learning opportunities, which could be one of the reasons for the drop in performance in the PHSL 2004A course in 2021 compared to previous cohorts that enrolled at the university and studied the course during pre-COVID-19 pandemic years. In their study on student academic achievement in the non-lecture physiology topics, Varachotisate et al. (2023) reported summative scores that were 4.80% ± 0.92% higher in the pre-COVID-19 year than in the COVID-19 year, and the student cohort opined that compared to the traditional face-to-face on-site and in-class learning, online learning reduced peer-to-peer and learner-educator interaction, albeit the students preferred the latter. It is also our contention that virtual online asynchronous content delivery and learning took away the benefits of peer-to-peer and peer-to-educator interactions, resulting in the drop in the pass rate in the PHSL 2004A course in 2021. In addition to the possible shock to students caused by the sudden change from the traditional lecture theatre approach to teaching and learning to online learning, members of the academy may have lacked the requisite competencies to package subject matter content in a user-friendly manner that facilitated students’ self-directed learning.

The discernible disparity in academic performance between MBBCh and BHSc students in the PHSL 2004A course during the academic year 2021, despite a decrease in pass rates compared to pre-COVID benchmarks, raises several questions. One important factor to consider is that the admission requirements for the MBBCh programme are typically more rigorous than those for BHSc counterparts, despite the shared curriculum in the first 2 years of study. This admission standards discrepancy raises important questions about the fairness of academic assessments within the common educational framework. Also, the current configuration of the PHSL 2004A curriculum raises questions about its suitability for BHSc, which is a general degree, and MBBCh, which is a professional degree. The students enrolled in these programmes have different educational goals. As a result, it is important to consider whether the curriculum is calibrated in a way that is fair and equitable for both programmes.

### Limitations and implications for future studies

The study retrospectively examined factors influencing academic performance and progression outcomes among MBBCh and BHSc students enrolled in the PHSL2004A course during the COVID-19-induced asynchronous learning environment. While the authors acknowledge that asynchronous learning may have impacted student performance, this aspect was not investigated to determine its specific role. Additionally, the disparities in student performance across admission categories warrant further research to explore the unique variations these categories independently contribute, both before and after COVID-19 pandemic. Such insights could provide evidence to inform potential revisions to the admission policy, aiming to enhance the academic success of students from equity groups.

## Conclusion

Our study provides valuable insights into the impact of programme affiliation, race and admission categories on academic performance and progression outcomes among medical (MBBCh) and health science (BHSc) students in the PHSL2004A course. The performance of MBBCh students consistently surpassed that of their BHSc counterparts, but the influence of living arrangements on progression outcomes was not found to be significant. Unfortunately, the shift to online learning as a result of the COVID-19 pandemic may have influenced student performance. Targeted support programmes and improvements in virtual learning experiences are crucial for addressing disparities and promoting equity. Through the implementation of these measures, we can strive to create a more level playing field and improve academic achievement in medical and health science education.
